# Reply to: Concerns about using a digital mask to safeguard patient privacy

**DOI:** 10.1038/s41591-023-02435-z

**Published:** 2023-07-18

**Authors:** Yahan Yang, Junfeng Lyu, Ruixin Wang, Feng Xu, Qionghai Dai, Haotian Lin

**Affiliations:** 1 State Key Laboratory of Ophthalmology, Zhongshan Ophthalmic Center, Sun Yat-sen University, Guangdong Provincial Key Laboratory of Ophthalmology and Vision Science, Guangdong Provincial Clinical Research Center for Ocular Diseases, Guangzhou, China; 2 School of Software and BNRist, Tsinghua University, Beijing, China; 3 Beijing Laboratory of Brain and Cognitive Intelligence, Beijing Municipal Education Commission, Beijing, China; 4 Department of Automation and BNRist, Tsinghua University, Beijing, China; 5 Hainan Eye Hospital and Key Laboratory of Ophthalmology, Zhongshan Ophthalmic Center, Sun Yat-sen University, Haikou, China; 6 Center for Precision Medicine and Department of Genetics and Biomedical Informatics, Zhongshan School of Medicine, Sun Yat-sen University, Guangzhou, China

**Keywords:** Medical research, Signs and symptoms, Biomedical engineering, Social sciences

replying to 10.1038/s41591-023-02439-9

Due to the entanglement of disease-relevant features with identifiable features of patients when using facial imaging, our original article developed a new technology, called a digital mask (DM), which is based on three-dimensional reconstruction and deep learning algorithms to minimize the risk of patient identification while enabling clinical diagnoses^1^. For validation, we designed experiments involving quantitative evaluation, diagnostic comparisons, reidentification by humans, AI-powered reidentification, and investigation of the level of patient satisfaction with the DM. Potential AI-powered attacks can be in various forms and as the DM is a new technique, such attacks cannot be fully considered in an initial publication. Therefore, we prioritized simulating attacks using publicly available face recognition systems, which are typically trained on RGB (red, green and blue) images. While we appreciate the work of Meeus et al. in proposing the possibility of a Mask2Mask attack for further testing of the DM technique, we think that the methodology used by Meeus et al. is not very relevant to the real-world situation.

Specifically, we think that their experiments are not rigorous enough in three aspects. First, the dataset used by Meeus et al. is not comparable to the one used in the original publication. Meeus et al. used retrospectively selected images of people in a non-medical setting, whereas the database we used contained images that had been prospectively collected in clinical settings, which are more suitable for clinical validation. Second, Meeus et al.’s Mask2Mask experiments are based on the assumption that both the algorithm and the face model (the parametric model that represents a three-dimensional face as shape and motion vectors) are known by the attackers, so that the masks in the query image and in the database are the same (a within-mask attack). However, in a real-world application, although the algorithm would be public, the face model can be made private for each institution, such that the most likely type of attack is a cross-mask attack, rather than a within-mask attack. Third, in the experimental setup of the Mask2Mask attack, the masks used for the query and database images were derived from the same video, which is not feasible in reality, because the original video of the clinical examination is private and attackers would be able to access only the DM video generated from the original video. We note that this is the major premise of the attack simulation: if attackers were able to access the original video, there would be no need for a Mask2Mask attack. Therefore, for a more realistic Mask2Mask attack, the query and database masks in the Mask2Mask setup should be generated from different videos.

Thus, the results of Meeus et al. show only attackers applying the same algorithm and face model to the same original video can achieve an effective attack. However, even with such strict requirements, the rank-1 accuracy reported by Meeus et al. was only about 50% when tested on a database of 555 individuals. In real-world applications, the accuracy of such an attack would be expected to be lower when using databases containing a larger number of individuals. We are currently undertaking more systematic Mask2Mask experiments to explore these issues.

It is important to reiterate our research motivation. First, the use of patient facial images in clinical diagnosis and academic communication is commonly used for improving diagnostic efficiency. With the development of digital medicine technologies, there is widespread demand for clinical imaging of patients’ faces for diagnosis^2–4^, medical journals feature clinical images of patients for sharing clinical cases and promoting medical education, and patient examination videos are shared on social media. In this context, an alternative is needed to reduce the privacy risk of using the original facial images.

As noted in the Discussion section of our original article, we aimed to reduce identification risks rather than achieve absolute de-identification. Due to the high entanglement of disease and identity features, our intention is to develop tools to safeguard patient privacy as much as possible, without compromising the need for the clinician to reach a diagnosis. In future research, we will optimize our technique to mitigate potential attacks, such as adding adversarial noise to the DM images to further decrease the risk of AI-powered reidentification^5–7^.

Before the large-scale application of this type of technology, all stakeholders, including patients, health institutions and institutional review boards, scientists and scientific communities, as well as regulatory and law enforcement agencies, must collaborate closely to maximize the protection of patient privacy^8,9^. Informed consent should be obtained from patients, which entails informing them of the benefits and potential attack risks associated with the technology. Moreover, as we stated in our original article, risks should be mitigated by formulating future relevant rules.

We thank Meeus et al. again for their interest in our article and their insights, and we agree that facial anonymization techniques need rigorous testing. Before the large-scale application of privacy-protection technologies, further research will be needed to ensure their rapid development.
